# Ratio between *Lactobacillus plantarum* and *Acetobacter pomorum* on the surface of *Drosophila melanogaster* adult flies depends on cuticle melanisation

**DOI:** 10.1186/s13104-021-05766-7

**Published:** 2021-09-08

**Authors:** Vladislav Mokeev, Justin Flaven-Pouchon, Yiwen Wang, Nicole Gehring, Bernard Moussian

**Affiliations:** 1 University of Tübingen, Interfaculty Institute of Cell Biology, Section Animal Genetics, Auf der Morgenstelle 15, 72076 Tübingen, Germany; 2 School of Pharmaceutical Science and Technology, Tianjin University, Tianjin, 300072 China; 3 Université Côte d’Azur, Parc Valrose, 06108 Nice CEDEX 2, France

**Keywords:** Microbiome, Bacteria, Insect, *Drosophila*, Cuticle

## Abstract

**Objectives:**

As in most organisms, the surface of the fruit fly *Drosophila melanogaster* is associated with bacteria. To examine whether this association depends on cuticle quality, we isolated and quantified surface bacteria in normal and melanized flies applying a new and simple protocol.

**Results:**

On wild flies maintained in the laboratory, we identified two persistently culturable species as *Lactobacillus plantarum* and *Acetobacter pomorum* by 16S rDNA sequencing. For quantification, we showered single flies for DNA extraction avoiding the rectum to prevent contamination from the gut. In quantitative PCR analyses, we determined the relative abundance of these two species in surface wash samples. On average, we found 17-times more *A. pomorum* than *L. plantarum*. To tentatively study the importance of the cuticle for the interaction of the surface with these bacteria, applying Crispr/Cas9 gene editing in the initial wild flies, we generated flies mutant for the *ebony* gene needed for cuticle melanisation and determined the *L. plantarum* to *A. pomorum* ratio on these flies. We found that the ratio between the two bacterial species reversed on *ebony* flies. We hypothesize that the cuticle chemistry is crucial for surface bacteria composition. This finding may inspire future studies on cuticle-microbiome interactions.

**Supplementary Information:**

The online version contains supplementary material available at 10.1186/s13104-021-05766-7.

## Introduction

Bacteria populate the surface of many organisms. While skin bacteria are well analysed in vertebrates including humans [1, 2], surface bacteria-insect interactions have been largely neglected to date. Most of data on surface bacteria come from studies in ants such as *Camponotus femoratus* and *Crematogaster levior*. In ant colonies, surface bacteria are considered to be involved in protection against fungal infection [3, 4]. A few data are available on bacteria on the surface of the fruit fly *Drosophila melanogaster*. The most common surface bacteria in this species belong to the genera *Lactobacillus* and *Acetobacter* [5]. The role of surface bacteria in *D. melanogaster* has not been studied and remains speculative.

The parameters on the insect defining bacteria-insect surface association are largely unknown. It is conceivable that microorganisms interact with components of the cuticle that is a stratified extracellular matrix composed of chitin, proteins, catecholamines and lipids [6]. Especially, the components of the surface called envelope including waxes and cuticular hydrocarbons (CHCs) [7] may be used as a substrate for bacterial attachment and/or for nutrition. In addition, this interaction may also depend on the inner-cuticle chemical environment including water content that in turn, at least partly, depends on the hardening and melanisation degree of the cuticle that involves a well-studied cascade of reactions catalysed by cytoplasmic and extracellular enzymes [8].

In the present work, we have designed a protocol for surface bacteria isolation and relative quantification in *D. melanogaster*. In a pilot experiment, we show that the bacterial composition depends on cuticle melanisation.

## Main text

### Materials and methods

#### Fly work

Tübingen^2018^ field flies deriving from seven founder flies caught in Tübingen were kept under laboratory conditions (22 °C, 50–70% air humidity) in vials with artificial diet consisting of corn meal, agar, beet sugar, propionic acid, dry yeast and Nipagin M. For fluorescein feeding, fluorescein sodium salt (Sigma-Aldrich) was mixed with fresh baker’s yeast added to the vials.

#### Isolation of surface bacteria

Flies were individually rubbed against the surface of a sterile agar plate (China Blue, ECI, EMB, LB, BHI and MRS, Sigma-Aldrich) inside laminar conditions using sterile forceps and incubated for 1–5 days aerobically. Among mixed populations of different microorganisms, individual colonies were isolated and sub-cultured twice to ensure purity. Bacteria were characterized morphologically using a light microscope and identified by 16S rDNA analysis.

#### Molecular biology

DNA template was prepared from individual bacterial colonies. 16S rDNA amplification was carried out according to a standard protocol by PCR using the universal primers 27F (5ʹ-AGAGTTTGATCCTGGCTCAG-3ʹ) and 1492R (5ʹ-GGTTACCTTGTTACGACTT-3ʹ) [9]. PCR products were purified using the GenElute™ PCR Clean-Up Kit (Sigma-Aldrich) and sequenced (Macrogen). Sequences were aligned to sequences of the NCBI database using BlastN.

For quantitative PCR (qPCR) experiments, single flies were immobilised with forceps and spilled with Tris–EDTA (pH8.0) containing 200 ng/µl Proteinase K avoiding the rectum. The wash solutions of 20 flies were combined, incubated at 65 °C for 30 min and frozen at − 20 °C. After thawing and centrifugation, 5 µl of this solution was used in a 10 µl reaction solution containing 1 µl of each species-specific primer and 2 µl of the FastStart Essential DNA Green Master solution (Roche). For species-specific qPCR, the primers pREV (5ʹ-TCGGGATTACCAAACATCAC-3ʹ) and pLanF (5ʹ-CCGTTTCTGCGGAACACCTA-3ʹ) to amplify *recA* (318 bp) in *Lactobacillus plantarum* [10] and PASTEU-F (5ʹ-TCAAGTCCTCATGGCCCTTATG-3ʹ) and PASTEU-R (5ʹ-TCGAGTTGCAGAGTGCAATCC-3ʹ) to amplify 130 bp of the 16S rDNA loci of *Acetobacter* species including *A. pomorum* and *A. pasteurianus* were used [11].

#### qPCR data analysis and statistics

After determination of the Cq values of the bacterial amplicons on a Roche LightCycler Nano with the respective software, we determined the fold-differences between the expression levels of each species by calculating 2^(highestCq−lowestCq)^ (i.e., 2^(Cq*Lpla*−Cq*Apom*)^ or 2^(Cq*Apom*−Cq*Lpla*)^). In extracts from Tübingen^2018^ flies (n = 5), the Cq values of the *L. plantarum* amplicon were always higher than the Cq values of the *A. pomorum* amplicon, while in extracts from *ebony* flies (n = 6), in all but one case, the Cq values of the *A. pomorum* amplicon were always higher than the Cq values of the *L. plantarum* amplicon; in the one outlier case, the fold-difference was almost one. To account for this qualitative shift between *A. pomorum* versus *L. plantarum* abundance, we reversed the calculated fold-difference by multiplication with − 1 when the *L. plantarum* Cq was lower than the *A. pomorum* Cq in our statistical analyses. As normal distribution of data could not be reasonably assumed (n = 5 and 6), data were analysed by the non-parametric Mann–Whitney *U*-test.

#### Gene editing

To mutate the *ebony* locus in Tübingen^2018^ flies, gene editing according to the Crispr/cas9 method was applied. We used the published gDNA (oligos: 5ʹ-GCGTTTAGTCGCAAAGAAGAA-3ʹ and 5ʹ-TACTGCCCGAGGTGTAGAGC-3ʹ) directed against the *ebony* gene sub-cloned in the pCDF3 vector [12]. This construct (550 ng/µl TE buffer) was injected into pre-blastoderm embryos together with 250 ng/µl of Cas9 protein (New England Biolabs). To identify mutant *ebony* alleles, the respective flies were crossed to flies segregating the known *ebony*^*1*^ allele. Stocks of dark flies were established. Homozygous *ebony* mutant flies (*ebony*^*cc1, 3* or *4*^) were sequenced to identify the mutation.

### Results and discussion

#### Isolation and quantification of *D. melanogaster* surface bacteria

We have developed a simple protocol to isolate and quantify surface bacteria from adult *D. melanogaster* by single fly showering for DNA extraction and subsequent quantitative PCR (qPCR). During the wash procedure, we avoided the contact of the wash solution with the rectum thereby preventing contamination with gut microbes. This protocol differs substantially from a protocol published recently on ant surface bacteria identification [13]. In that case, whole ants were stirred in microtubes for DNA extraction. In a strict sense, this protocol cannot exclude gut microbe contamination in the wash solution. We assume that our simple protocol is applicable on any insect species.

#### *Lactobacillus plantarum* and *Acetobacter pomorum* are the major culturable bacteria on the fly surface

**Fig. 1:**
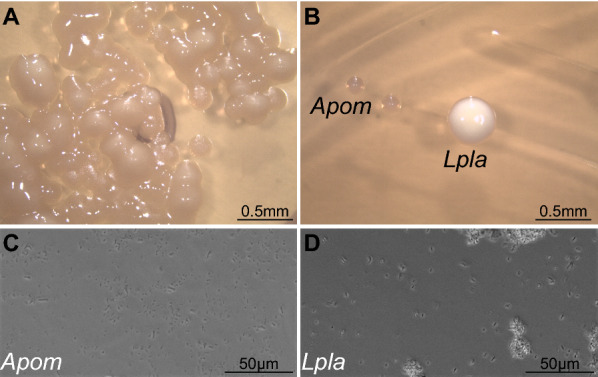
*Lactobacillus plantarum* and *A. pomorum* are present on the surface of *D. melanogaster*. Upon streaking *D. melanogaster* on an MRS plate. Two types of heaps of bacteria were observed (**A**). We isolated single colonies that were white or beige (**B**). After sequencing the 16S rDNA, the white colonies were identified as *L. plantarum* (Lpla) and the beige colonies as *A. pomorum* (Apom). Under the microscope, both bacteria are rode shaped [*L. plantarum* (**C**). *A. pomorum* (**D**)]

To isolate and characterise surface bacteria, we streaked the dorsum of living Tübingen^2018^ flies on different media including MRS (DeMan, Rogosa, Sharpe). To exclude gut-derived bacteria, we avoided contacting the rectum with the medium. Persistently, in independent experiments, we observed two types of colonies on MRS plates (Fig. 1). The colonies were round and white or yellowish. Under the light microscope, bacteria from both colonies showed a rode shape (Fig. 1). To determine the species, we amplified the 16S rDNA locus using universal primers and sequenced the amplicon. Alignment of the amplified sequences with sequences from the NCBI nucleotide database revealed that the 16S rDNA sequence from bacteria forming white colonies was similar to the respective sequence from *Lactobacillus plantarum* (Table 1), while the 16S rDNA sequence from bacteria forming yellow colonies was similar to the respective sequence from *Acetobacter* species including *A. pomorum* and *A. pasteurianus* (Table 1). To distinguish between these two species, we determined the sequence of the *groEL* gene. The sequence amplified from our bacteria was more similar to the *groEL* sequence of *A. pomorum* than to the respective sequence of *A. pasteurianus*.

**Table 1:** Identification of the bacterial species

Locus	Sequence length	Identity (%)	Species
*16S rDNA*	853 bp	98.25	*Lactobacillus plantarum*
		98.02	*Lactobacillus pentosus*
*16S rDNA*	982 bp	99.80	*Lactobacillus plantarum*
		99.69	*Lactobacillus pentosus*
*16S rDNA*	940 bp	99.25	*Acetobacter pomorum*
		99.25	*Acetobacter pasteurianus*
*16S rDNA*	1050 bp	100	*Acetobacter pomorum*
		100	*Acetobacter pasteurianus*
*groEL*	560 bp	98.57	*Acetobacter pomorum*
		97.96	*Acetobacter pasteurianus*

The sequence of the 16S rDNA locus suggests that the white colonies shown in Fig. 1 are *L. plantarum*. This sequence is not sufficient to determine the species of the beige colonies. Based on the *groEL* sequence. We assume that these bacteria are *A. pomorum*

Both species had been found to be present in the *D. melanogaster* gut [14]. In the gut, *L. plantarum* was reported to promote growth by interfering with the insulin and ecdysone signalling pathways on poor-condition medium [15]. In another work, it was found that, by contrast, intestinal *L. plantarum* had a negative effect on *D. melanogaster* life span [16]. We presume that *L. plantarum* on the cuticle surface does not contribute to any of these effects as the gut and surface micro-environments are fundamentally different. Indeed, while in the gut these bacteria live under anaerobic conditions, on the fly surface, they rather face aerobic conditions. Supposedly, their physiology and, by consequence, their role changes accordingly. For instance, aerobic but not anaerobic cultures of *L. plantarum* produce H_2_O_2_ [17]. At the cuticle surface, H_2_O_2_ might oxidise CHCs and thereby modify the barrier function of this layer. A second possible function of *L. plantarum* on the cuticle surface is partner attraction and to promote crowding. Indeed, *D. melanogaster* has been shown to be attracted by yet unidentified volatile compounds of *L. plantarum* [18].

#### The fly surface is not soiled by faeces

*Lactobacillus plantarum* and *A. pomorum* are also present in the gut suggesting that their presence on the fly surface may originate from faeces. To verify whether the surface of flies contains excreted material, we fed flies with fluorescein and imaged their surface by fluorescence microscopy (Additional file 1: Figure S1). Only very little fluorescence signal was detected on the fly surface. We conclude that contamination of the surface by faeces is a very rare event and therefore probably negligible. This observation, nevertheless, allows the hypothesis that faecal bacteria might be the actual source of the surface populations.

#### Relative quantification of bacteria by qPCR

**Fig. 2:**
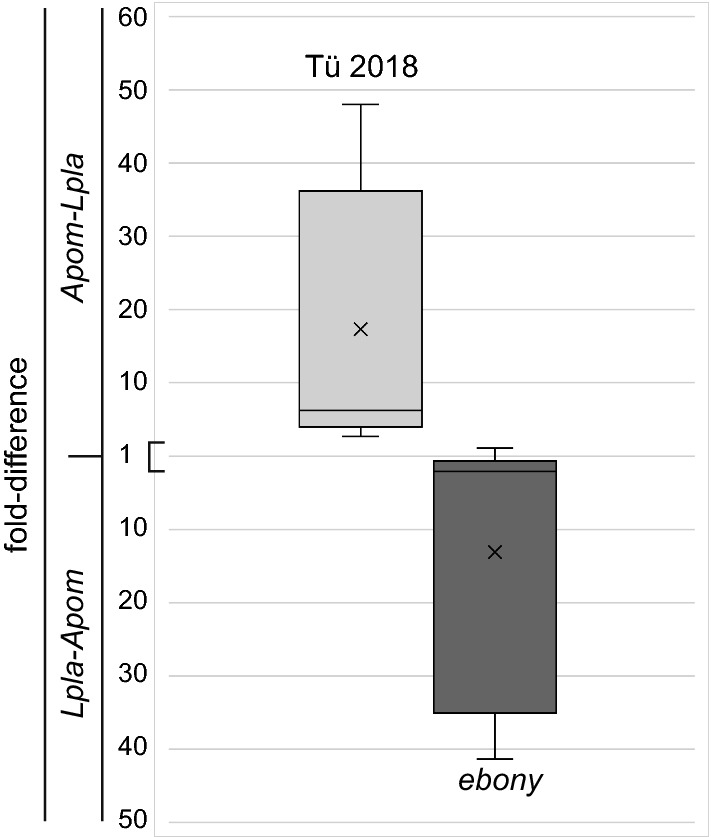
The ratio between *L. plantarum* and *A. pomorum* depends on the fly genotype. Applying qPCR, we detected *L. plantarum* and *A. pomorum* in the wash solution of fly surfaces in independent experiments using genus-specific primers (1–3). In *ebony* mutant flies (*ebony*^*cc1. 3 & 4*^) that derive from Tübingen^2018^ flies (Tü 2018) by gene editing, the relative fold-difference (y-axis) between *L. plantarum* and *A. pomorum* is reversed compared to the situation in the original flies. Data are shown as boxplots (n = 5 for Tübingen^2018^. n = 6 for *ebony* flies). Central traits represent the median, crosses the mean. Boxes indicate first and third quartile and whiskers represent the range. The broad data range is probably due to the genetic variation of the fly populations that derive from several founders. Data were analysed using the non-parametric Mann–Whitney *U*-test with the null hypothesis (H0) that the two populations “Tübingen^2018^” and “*ebony*” are not different. With α = 0.05 and U = 30. The p-value for this test is 0.004 allowing to refute H0. Applying the parametric Student’s *T*-test, we obtain a p-value of 0.013 (data now shown) suggesting a significant difference between the data of the two populations. We consider this test, however, as inappropriate as we cannot assume a normal distribution of the data

Isolation and cultivation of bacteria from the fly surface on media plates does not allow relative quantification as standardisation of bacterial transfer from flies to the plate is not possible. Therefore, we determined the fold-differences between *L. plantarum* and *A. pomorum* indirectly using species-specific primers in qPCR experiments. We compared their abundance on Tübingen^2018^ flies (Fig. 2). In mean, there were 17 times more *A. pomorum* than *L. plantarum* on these flies.

In order to test the influence of the cuticle on the load of *L. plantarum* and *A. pomorum*, we introduced mutations in the *ebony* gene of Tübingen^2018^ flies that codes for β-alanyl-dopamine (NBAD) synthase involved in cuticle melanisation [8]. Three independent mutations in the *ebony* gene (*ebony*^*cc1*^, *ebony*^*cc3*^, *ebony*^*cc4*^) were recovered. The respective homozygous mutant flies that are darker than wild-type flies are viable. We determined the fold-difference between *L. plantarum* and *A. pomorum* on the surface of *ebony* flies by qPCR (Fig. 2). We found that compared to Tübingen^2018^ control flies, *L. plantarum* were more abundant than *A. pomorum* on *ebony* flies. We conclude that their load depends on Ebony and probably on melanisation. It remains to be shown whether Ebony and melanisation either promote *L. plantarum* or inhibit *A. pomorum* growth. Classically, according to the melanism-desiccation hypothesis, enhanced melanisation has been considered as a response to dry environment to prevent desiccation [7]. For instance, in the melanic drosophilid *D. kikkawai* higher abdominal melanisation correlates with enhanced desiccation resistance [19]. However, there are cases reported that contradict this hypothesis [20, 21]. Desiccation resistance, for example, did not correlate with the body colour intensity in *D. melanogaster* field populations in India [21]. Thus, melanisation is probably a trade-off trait not only dictated by humidity conditions. Based on this assumption, we speculate that Ebony-driven melanisation may also be involved in controlling the interaction between the fly body and bacteria conferring a yet unknown advantage. Alternatively, Ebony may have a function in the differentiation of the envelope and the surface CHCs. Indeed, recently, it was found that longer chain CHCs prevailed in *ebony* mutant females [22]. This suggests that *L. plantarum* and *A. pomorum* differ in their preference on CHC environment. In summary, these data support the view that the insect cuticle surface is not an inert substrate for bacteria.

## Limitations

The ratio between *L. plantarum* and *A. pomorum* on the surface of *D. melanogaster* changes depending on the genetic background suggesting that the insect-bacteria interaction may be under genetic control. The significance of this interaction is unclear as our conclusion relies only on the impact of a single gene i.e., *ebony* on the insect-bacteria interaction. More work is needed in this direction.

We should point out that the flies used in this work were kept under laboratory conditions. Hence, it is unclear whether our work reflects the situation in the field.

A major uncertainty in this work concerns the bacterial species. The amplified *A. pomorum* 16S rDNA sequence is 100% identical to the respective sequence in *A. pasteurianus* [11]. The provisional identification of *A. pomorum* is based on the *groEL* sequence. The *groEL* sequence determined in this work is, however, not identical to the *A. pomorum groEL* sequence from the NCBI database. Thus, it is well possible that the *Acetobacter* species isolated in this work is neither *pomorum* nor *pasteurianus* but a third yet unknown species not present in the sequence databases. Additional analyses are needed to clarify this issue.

## Supplementary Information

**Additional file 1: Figure S1.** There are only little faeces on the fly surface. The surface of flies fed with yeast supplemented with fluorescein did not show abundant fluorescence signal (arrows). To visualize fluorescein traces on the fly surface, flies were anesthetized with CO_2_ and viewed with the Nikon AZ100 using fluorescence microscopy mode with a LED light source and a F36-525 HC-set EGFP filter. Bacterial colonies in Fig. 1 were observed and imaged on a Leica EZ4 stereomicroscope with in-built camera using the software LAX. Bacterial cells were viewed on a Nikon Ti2 microscope using phase contrast microscopy with a S Plan Fluor ELWD 40 × Ph2 ADM objective.

## Data Availability

All data are presented in the manuscript. Upon request, fly stocks will be shared by the corresponding author.

## Abbreviations

Apom: *Acetobacter pomorum*
bp: Base pairs
CHC: Cuticular hydrocarbon
Crispr/Cas9: Clustered regularly interspaced short palindromic repeats/CRISPR-associated protein 9
gDNA: guide DNA
groEL: 60 KDa chaperonin
Lpla: *Lactobacillus planatrum*
qPCR: quantitative polymerase chain reaction
16S rDNA: 16S ribosomal DNA
