# Supplementary figures and images for: Ratio between Lactobacillus plantarum and Acetobacter pomorum on the surface of Drosophila melanogaster adult flies depends on cuticle melanisation
Source: BMC Res Notes. 2021 Sep 8;14:351. doi: 10.1186/s13104-021-05766-7 (PMC8425098; doi:10.1186/s13104-021-05766-7)

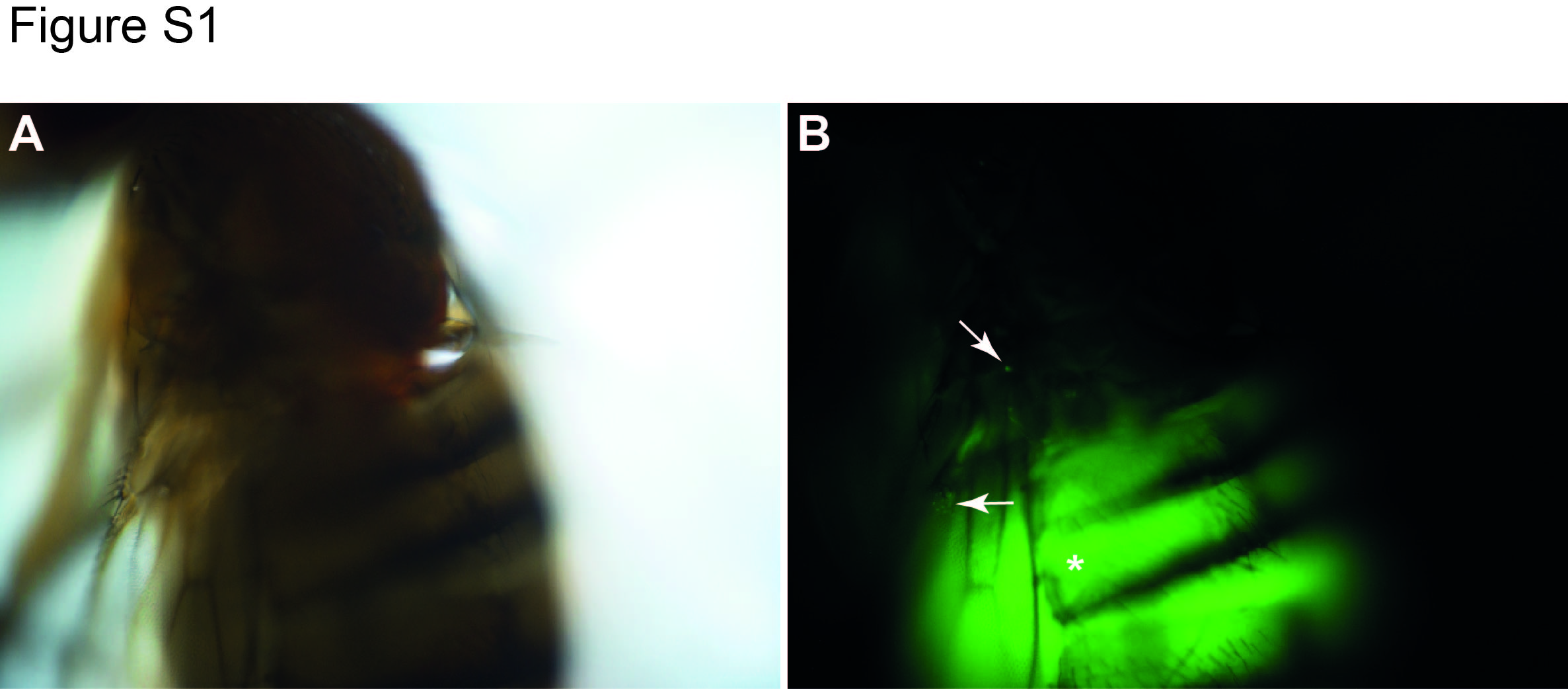

Supplement: Supplementary file 1 — Additional file 1: Figure S1. There are only little faeces on the fly surface. The surface of flies fed with yeast supplemented with fluorescein did not show abundant fluorescence signal (arrows). To visualize fluorescein traces on the fly surface, flies were anesthetized with CO2 and viewed with the Nikon AZ100 using fluorescence microscopy mode with a LED light source and a F36-525 HC-set EGFP filter. Bacterial colonies in Fig. 1 were observed and imaged on a Leica EZ4 stereomicroscope with in-built camera using the software LAX. Bacterial cells were viewed on a Nikon Ti2 microscope using phase contrast microscopy with a S Plan Fluor ELWD 40 × Ph2 ADM objective. [file 13104_2021_5766_MOESM1_ESM.jpg]
